# Tumour suppressive long non‐coding RNA AFDN‐DT inhibits gastric cancer invasion via transcriptional regulation

**DOI:** 10.1111/jcmm.14988

**Published:** 2020-01-24

**Authors:** Yuexing Lai, Ping Xu, Jing Wang, Kai Xu, Lin Wang, Yuchen Meng

**Affiliations:** ^1^ Department of Gastroenterology Songjiang Hospital Affiliated to Shanghai Jiaotong University School of Medicine (Preparatory Stage) Shanghai China; ^2^ Department of Gastroenterology Songjiang Hospital Affiliated to Nanjing Medical University Shanghai China; ^3^ Department of Central laboratory Songjiang Hospital Affiliated to Shanghai Jiaotong University School of Medicine (Preparatory Stage) Shanghai China

**Keywords:** AFDN divergent transcript, gastric cancer, lncRNA, transcriptional regulation

## Abstract

Emerging evidence has revealed that dysregulation of lncRNA is associated with the initiation and progression of cancer. However, the function of these lncRNAs in cancer remains largely unexplored. Here, we reported that AFDN‐DT, an lncRNA that is repressed in gastric cancers (GC), functions as a tumour suppressor by inhibiting cell growth and metastasis through transcriptional repression of genes involved in metastasis. Using in vitro and in vivo models, we demonstrated that overexpression of AFDN‐DT inhibited the proliferation and metastasis of GC. We found that AFDN‐DT was located at the nucleus and interacted with the chromatin in gastric cells. Further, ChIRP‐seq experiments and RNA‐seq analysis revealed that AFDN‐DT directly bound to the promoter regions and regulated the expression of genes essential for malignant transformation. Moreover, we demonstrated that DNA hypermethylation could repress AFDN‐DT expression and treatment with DNA methylation inhibitors restored its expression. Collectively, the results of our study demonstrated the tumour suppressive role of AFDN‐DT in GC and elucidated the transcription regulatory role of tumour suppressive lncRNAs, which can serve as potential prognostic markers for GC.

## 1 INTRODUCTION

Gastric cancer (GC) is one of the most common malignant diseases and the second leading cause of cancer‐related deaths worldwide.^1^ Despite improved surgical techniques and chemotherapy, the overall survival of patients with GC remains unsatisfactory.^2^ A better understanding of the initiation, invasion, and metastasis of GC is required to improve its clinical outcome. Using high‐throughput sequencing, several genes with prognostic values have been studied extensively.^3^, ^4^, ^5^ However, these targets have been rarely applied for clinical diagnosis or therapeutic use due to the limited understanding of their functions in the tumorigenesis of GC.

Long non‐coding RNAs (lncRNAs) belong to the newly discovered category of regulatory RNAs with lengths greater than 200 nucleotides, and they do not encode any proteins.^6^, ^7^ Compared to the protein‐coding RNAs, lncRNAs are less conserved in different cell types and species. LncRNAs are regulators involve in multiple biological processes^6^, ^8^ including microRNA sponging,^9^ translational^10^ and transcriptional regulation^11^ and protein‐protein interactions.^12^ Moreover, many lncRNAs have shown good prognostic values in several forms of cancer.^6^, ^13^ Latest evidence on tumorigenesis has revealed that lncRNAs play a vital role in the tumorigenesis of many forms of cancers,^6^, ^13^ and several oncogenic lncRNAs, such as NEAT1,^7^, ^14^ MALAT1^15^, ^16^ and PVT1,^17^ are well established. In addition, certain other lncRNAs, such as GAS5^18^ and ZFAS1,^19^ have been shown to act as tumour suppressors during carcinogenesis.

AFDN divergent transcript (AFDN‐DT), also known as MLLT4 antisense RNA 1 (MLLT4‐AS1), is an lncRNA located upstream of the Afadin (AFDN) gene.^20^ AFDN, otherwise known as MLLT4 and AF6, has been identified as a fusion partner in Lysine Methyltransferase 2A gene (KMT2A).^21^ In our previous study, we showed that high levels of AFDN‐DT, but not AFDN, correlated with a favourable outcome in patients with GC^20^ and suggested that AFDN‐DT might be tumour suppressor, even though the mechanism of AFDN‐DT in GC remains completely unknown.

In the present study, we revealed that AFDN‐DT inhibits cell growth and invasion of GC cells. Moreover, RNA pull‐down experiments and RNA‐seq analysis revealed that AFDN‐DT directly interacts with transcription factors and histones, and it is involved in transcriptional regulation. Moreover, we demonstrated that DNA hypermethylation is mainly responsible for the repressed expression of AFDN‐DT, and treatment with DNA methylation inhibitors restored the expression of AFDN‐DT.

## 2 MATERIALS AND METHODS

### 2.1 Cell line and lentiviral transfection

HGC27 cell line was purchased from the Shanghai Cell Bank of the Chinese Academy of Sciences and cultured in RPMI 1640 (Hyclone) supplemented with 10% foetal bovine serum (FBS) (Biological Industries). The full‐length mRNA of AFDN‐DT was cloned into the Lenti‐CMV vector, and the lentivirus was produced as previously described.^22^ The lentivirus exhibiting AFDN‐DT overexpression was transfected into HGC27 cells, and the positively transduced cells were selected using puromycin. Cell growth was determined with the Cell Counting Kit‐8 (CCK‐8) (Dojindo) using the Multiskan FC Microplate spectrophotometer at OD450.

### 2.2 Quantitative reverse transcription PCR (RT‐qPCR)

Total RNA was extracted using TRIzol reagent (Life Technologies) according to the manufacturer's instructions. RT‐qPCR was performed using the AceQ Universal SYBR qPCR Master Mix (Vazyme) according to the manufacturer's instructions. The Primers used in this study are listed below. AFDN‐DT‐F: 5′‐CCCACTCCCTTTGTGTGTCT‐3′; AFDN‐DT‐R: 5′‐AGCTTCCCCTCGACGTTTAT‐3′; FOS‐F: 5′‐CCGGGGATAGCCTCTCTTACT‐3′; FOS‐R: 5′‐CCAGGTCCGTGCAGAAGTC‐3′; JUN‐F: 5′‐TCCAAGTGCCGAAAAAGGAAG‐3′; JUN‐R: 5′‐CGAGTTCTGAGCTTTCAAGGT‐3′; EGR1‐F: 5′‐GGTCAGTGGCCTAGTGAGC‐3′; EGR1‐R: 5′‐GTGCCGCTGAGTAAATGGGA‐3′; GAPDH‐F: 5′‐GGAGCGAGATCCCTCCAAAAT‐3′; GAPDH‐R: 5′‐GGCTGTTGTCATACTTCTCATGG‐3′.

### 2.3 Animal models

Twenty male BALB/c‐nu/nu mice (Shanghai SLAC Laboratory Animal Co., Ltd.) aged 6 weeks, were divided into two groups and were subcutaneously injected with 1 × 10^7^ AFDN‐DT or vector‐transfected HGC27 cells. The tumour volume was assessed 18 days after injection. All the mice were euthanized on day 34, and the tumour weight was evaluated.

### 2.4 Wound healing assay

AFDN‐DT or vector transduced HGC27 cells (5 × 10^5^ cells/well) were seeded into 6‐well plates (Corning). Wounds were made by scratching the adherent cells on the plate with a sterile 200 μl pipette tip (12 hours after seeding), replaced with fresh culture medium and then cultured for 24 hours. The migration ability was evaluated by analysing the migration of the cells into the wounded area.

### 2.5 Transwell migration assay

Cell migration was determined using the transwell membranes (Corning). Briefly, the upper transwell chamber was coated with 60 μL of Matrigel (BD) for 2 hours prior to the invasion assays. AFDN‐DT or vector transduced HGC27 cells (2 × 10^5^ cells/well) in serum‐free RPMI 1640 were seeded into the upper transwell chamber, and 600 μL of RPMI 1640 medium supplemented with 10% FBS was added to the lower chamber. After incubation for 24 hours, the cells that adhered to the upper surface of the membrane were removed. Meanwhile, the invaded or migrated cells, which adhered to the lower surface, were stained with 0.1% crystal violet and measured by optical microscopy.

### 2.6 RNA pull‐down experiments

RNA pull‐down experiments were performed using the Pierce™ Magnetic RNA‐Protein Pull‐Down Kit (#20164, Thermo Fisher) according to the manufacturer's instructions. The full‐length AFDN‐DT RNA was obtained using in vitro transcription with the T7 High Yield RNA Transcription kit (TR101, Vazyme), following which the RNA was biotinylated using the Pierce™ RNA 3´ Desthiobiotinylation Kit (# 20163, Thermo Fisher). LC‐MS/MS was performed at Shanghai Cutseq Bio‐medical Technology Co. Ltd.

### 2.7 RNA sequencing

RNA was extracted using TRIzol reagent (Life Technologies) according to the manufacturer's instructions. The RNA‐seq library was constructed using the VAHTS mRNA‐seq V2 Library Prep Kit for Illumina® (NR601, Vazyme) according to the manufacturer's instructions. The sequencing was performed using Illumina's Novaseq platform according to manufacturer's instruction. The raw RNA‐seq data have been deposited at the GEO database (GSE139326).

### 2.8 Chromatin isolation by RNA purification (ChIRP)

ChIRP was performed as previously described.^23^ The AFDN‐DT transduced HGC27 cells were fixed with 1% formaldehyde for 10 minutes and quenched with 1/20 volume of 2.5 mol/L glycine for 5 minutes. The cells were collected and washed twice with PBS and then resuspended in sonication buffer (20 mmol/L Tris‐HCl (PH 8), 2 mmol/L EDTA, 1% Triton X‐100, 150 mmol/L NaCl, 1% SDS). The samples were sonicated using the Bioruptor® Pico Sonication System (Diagenode, Belgium) for 10 cycles (30 seconds on/30 seconds off). After centrifugation for 10 minutes at 15 000 *g* at 4°C, the supernatants were collected and incubated with the biotinylated antisense DNA against AFDN‐DT at 4℃ overnight. The biotinylated oligos used for AFDN‐DT are the following: AFDN‐DT‐1:5′‐GCAGCAGCACCTAGTGGAGC‐3′; AFDN‐DT‐2:5′‐TGCCCATTTAGATCCAGCAG‐3′; AFDN‐DT‐3:5′‐TAGACCTAGCACCGCCCGTC‐3′; AFDN‐DT‐4:5′‐CGCCCATCGGACCCACCGCC‐3′; AFDN‐DT‐5:5′‐CCAGCAGCGCCCATTTGGAT‐3′; AFDN‐DT‐6:5′‐GCGAGCGCGGGGAGCGCAGG‐3′; AFDN‐DT‐7:5′‐TCAGAAAACATGACCCTTGA‐3′; AFDN‐DT‐8:5′‐CTACGTCTGCGAAGAATTGG‐3′; AFDN‐DT‐9:5′‐TCCTTGCTGTGCAGGCACCG‐3′; AFDN‐DT‐10:5′‐ACTTTGGACATCAGCAATCT‐3′; AFDN‐DT‐11:5′‐GAATGATTCACATTAATTTCG‐3′; AFDN‐DT‐12:5′‐ATTTAAGAATCATAGGTATT‐3′; AFDN‐DT‐13:5′‐AAGATGGTAGCATGTTTACC‐3′; AFDN‐DT‐14:5′‐CTCCTGACCTCGTGATCTGC‐3′. The antisense probes of each DNA were used as negative controls. Streptavidin magnetic beads were washed and added into the reaction mixture for 4 hours at 4℃. The beads were washed five times with the wash buffer (20 mmol/L Tris‐HCl (PH 8), 2 mmol/L EDTA, 1% Triton X‐100, 300 mmol/L NaCl, 0.2% SDS). The ChIRPed samples were eluted using biotin elution buffer and de‐crosslinked with Proteinase K at 65℃ overnight. The ChIRPed DNA was purified using the QIAquick PCR Purification Kit (Qiagen).

### 2.9 Bioinformatics analysis

All the sequenced reads were mapped to the hg38 genome using HISAT2,^24^ and the sequence alignment mapping (SAM) files were sorted using samtools.^25^ The expression of the genes was quantified using the htseq‐count,^26^ and the differentially expressed genes were identified using the DEseq2.^27^ Gene ontology analysis was performed using the DAVID Functional Annotation Bioinformatics Microarray Analysis database. Motif enrichment analysis was performed using the MEME suite.^28^

### 2.10 DNA methylation analysis

Information on DNA methylation and mRNA expression in the gastric tumour samples was obtained from TCGA cohort.^29^ DNA methylation was evaluated using the Illumina Human Methylation 450K BeadChip, and the normalized beta value of AFDN‐DT was used for quantification. The relative expression of AFDN‐DT was determined by RNA‐seq, and the RSEM values (RNA‐Seq by Expectation‐Maximization) were used for quantification.

### 2.11 Treatment with DNA methylation inhibitor

HGC27 cells were treated with or without the DNA methylation inhibitor, decitabine, at a final concentration of 5 μmol/L for 24 hours. DNA methylation was determined by meDIP‐qPCR according to the manufacturers’ instruction (#55009, Active motif). Primers used to determine the DNA methylation of the CpG island located at the promoter regions of AFDN‐DT are listed below. AFDN‐DT‐5mC‐F: 5′‐ CCAGACGGAACCCTAGCAC‐3′; AFDN‐DT‐5mC‐R: 5′‐ GCTCCACTAGGTGCTGCTG‐3′. Cell viability was determined using the CCK8 assay, and the relative expression of AFDN‐DT was determined by RT‐qPCR.

### 2.12 Statistical analysis

All experiments were independently performed in triplicate. GraphPad Prism 7.0a was used for the statistical analyses. Data are presented as the mean ± SD. Statistical significance was determined using Student's *t* test. A *P*‐value < .05 indicated statistical significance.

## 3 RESULTS

### 3.1 Inhibition of cell growth and invasion of GC cells by AFDN‐DT in vitro

**Figure 1:**
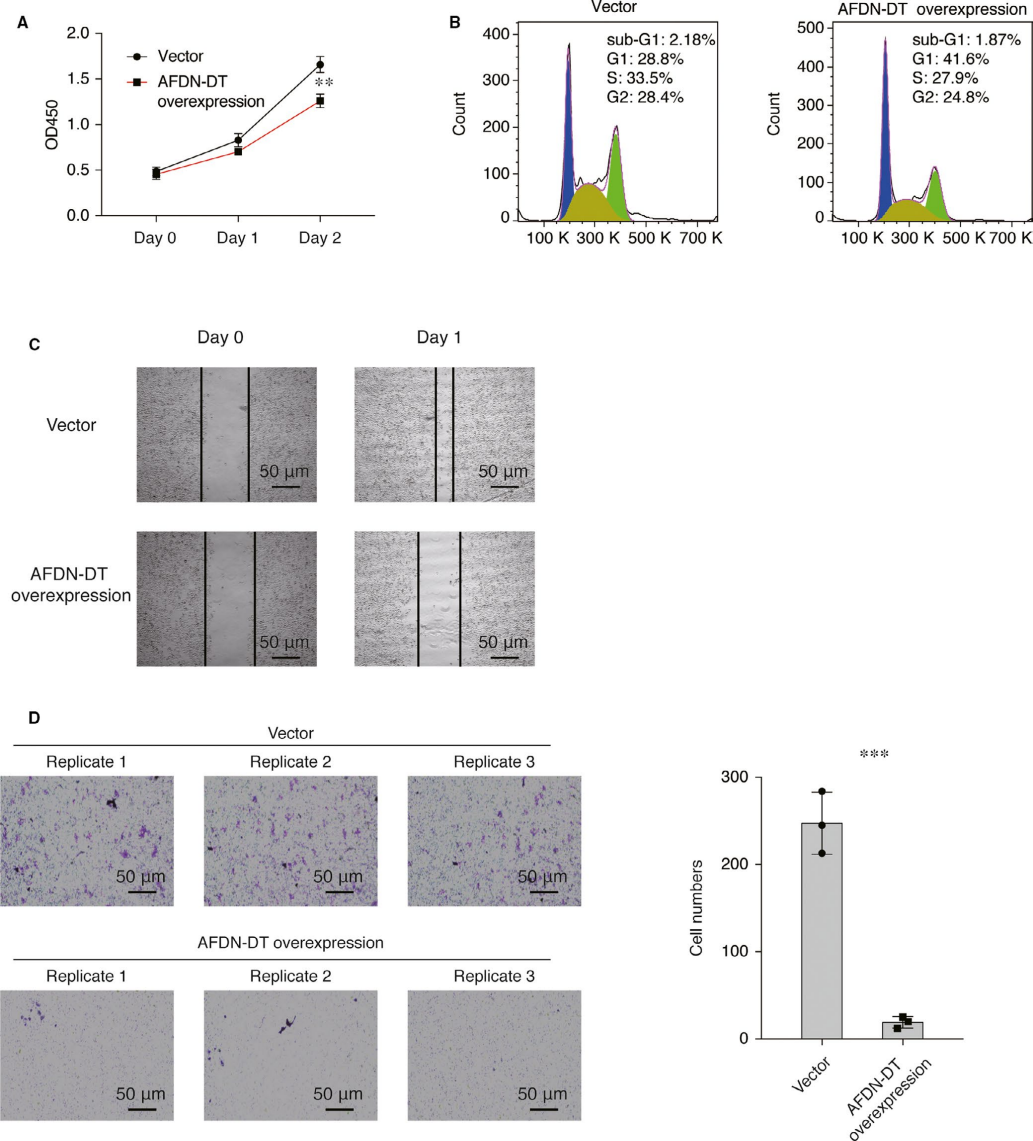
AFDN‐DT inhibits cell growth and invasion of gastric cancer cells. A, AFDN‐DT overexpression inhibits cell growth of HGC27 cells. The viability of cells transduced with AFDN‐DT or vector was determined at day 0, 1 and 2 using the CCK8 assays. B, AFDN‐DT overexpression induces cell cycle arrest. The cell cycle was determined by PI staining. C, AFDN‐DT overexpression inhibits migration of HGC27 cells. The wound healing ability was determined 24 h after scratching. D, AFDN‐DT overexpression inhibits invasion of HGC27 cells. The effects of AFDN‐DT on gastric cancer cell invasion were determined by transwell assays. Data represent the mean of three replicates ± SD, ***P < .0001

To determine the function of AFDN‐DT in GC, we firstly overexpressed AFDN‐DT in HGC27 cells derived from a patient with GC and found that overexpression of the AFDN‐DT inhibits the growth of the HGC27 cells (Figure 1A). Next, cell cycle analysis revealed that overexpression of AFDN‐DT induces G0/G1 cell cycle arrest in HGC27 cells, indicating a potential function of AFDN‐DT in cell growth regulation. We then performed wound healing experiments on the cells with or without AFDN‐DT overexpression and found that the migration ability was significantly decreased upon AFDN‐DT overexpression (Figure 1B). Further, we performed transwell invasion assays to determine the effect of AFDN‐DT on the metastasis. As shown in Figure 1C, the metastasized cells were significantly reduced with the overexpression of AFDN‐DT. Collectively, these experiments demonstrated that the AFDN‐DT induces cell growth inhibition and reduces the invasiveness of HGC27 cells.

### 3.2 In vivo tumour suppressive function of AFDN‐DT

**Figure 2:**
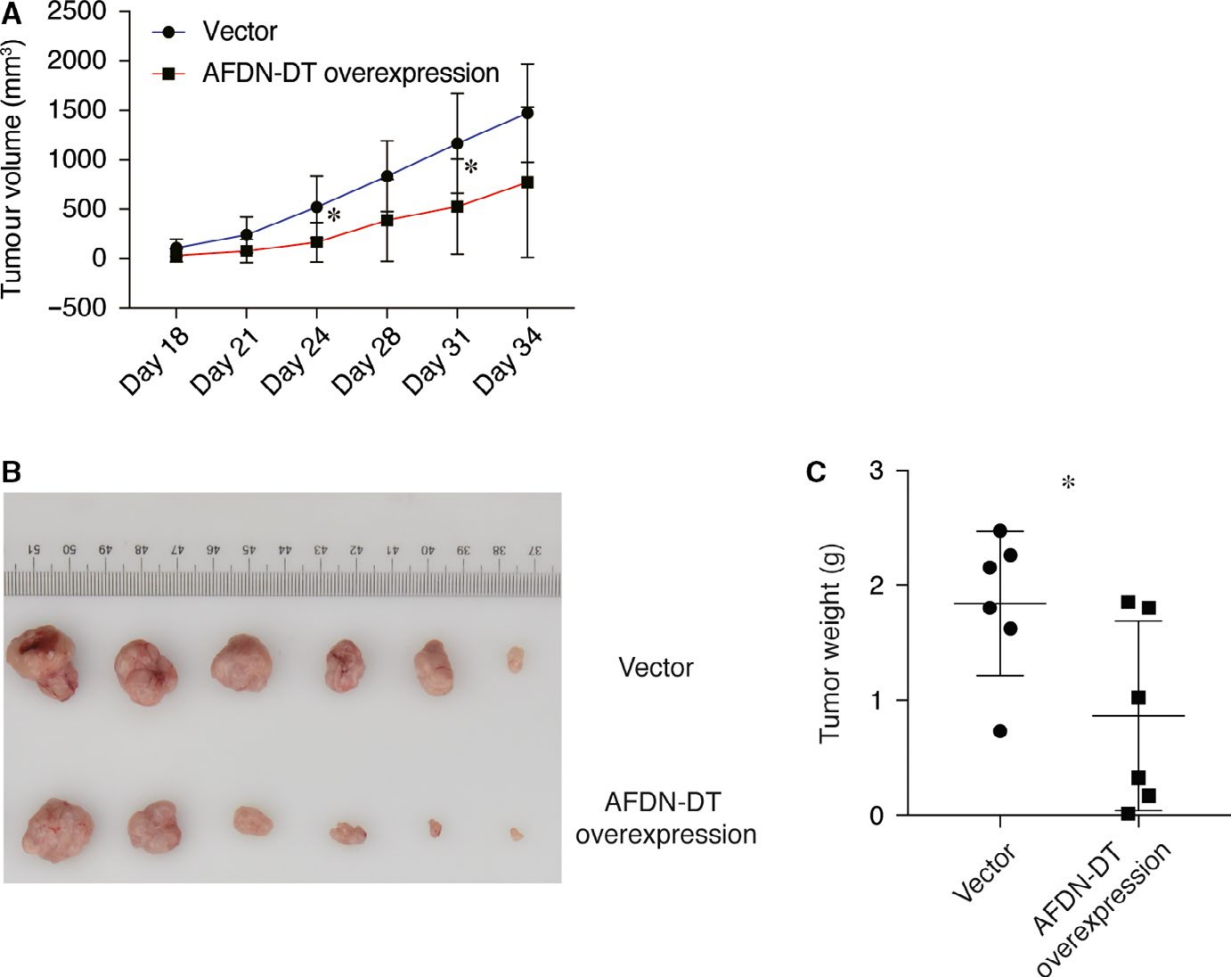
AFDN‐DT inhibits gastric cancer in vivo. A, AFDN‐DT inhibits tumour growth of HGC27 cells in nude mice. AFDN‐DT overexpressing or vector infected HGC27 cells were injected into nude mice (n = 6). Tumour growth curves with time series are displayed. B, Decreased tumour size with AFDN‐DT overexpression. C, Decreased tumour weight with AFDN‐DT overexpression. Data represent the mean of six replicates ± SD, *P < .01

To examine the in vivo function of AFDN‐DT further, we used a xenograft murine model by injecting the HGC27 cells with or without AFDN‐DT overexpression into the nude mice. As shown in Figure 2A, the cells with AFDN‐DT overexpression inhibited the growth of HGC27 cells. The tumour size and weight were significantly decreased in the mice injected cells transduced with AFDN‐DT overexpression plasmid (Figure 2B‐C). Collectively, these observations revealed the anti‐tumour activity of AFDN‐DT in vivo.

### 3.3 Interaction of AFDN‐DT with histone proteins in GC cells

**Figure 3:**
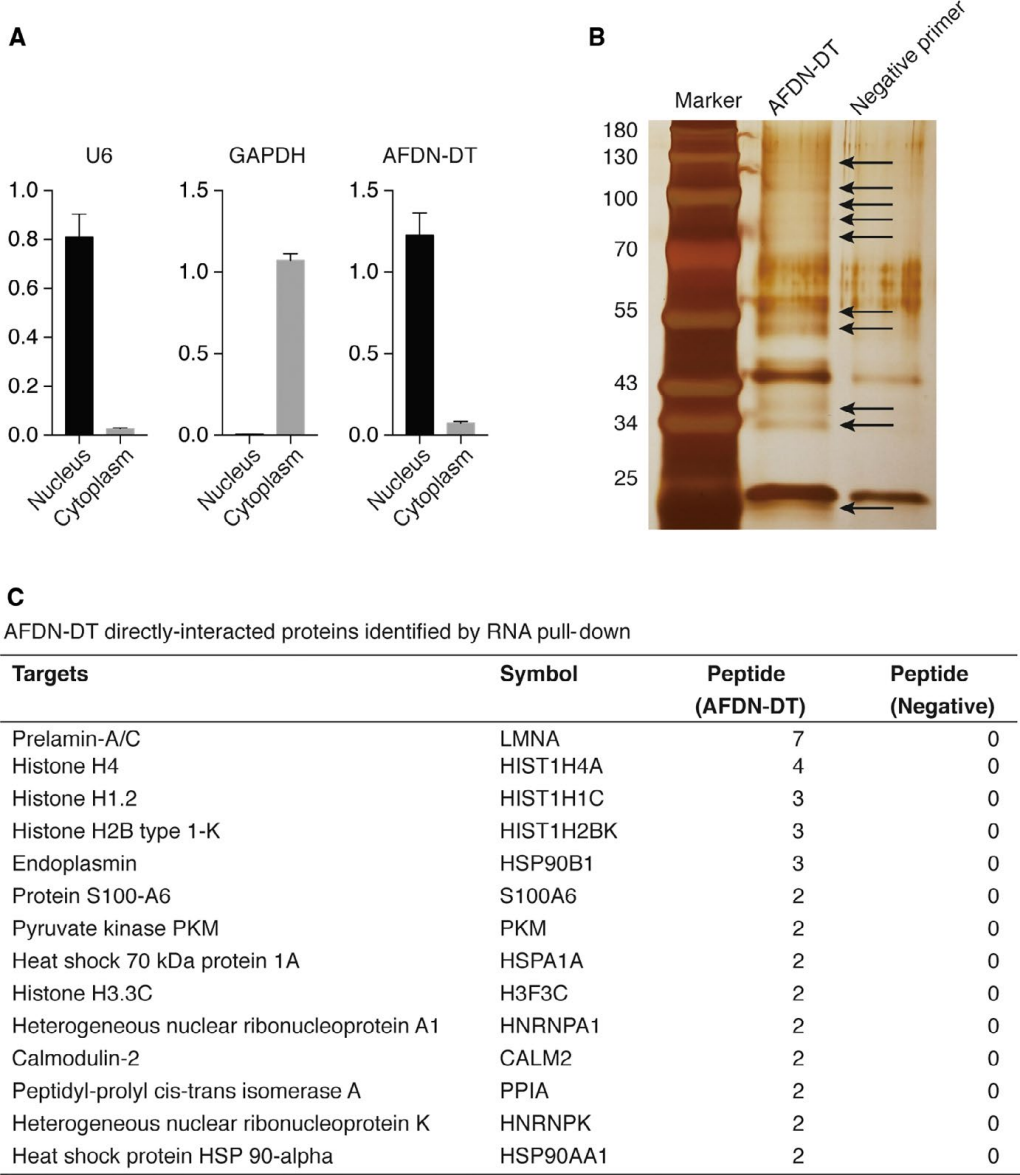
AFDN‐DT directly interacts with nucleosome‐related protein in gastric cancer cells. A, AFDN‐DT located in the nucleus regions. The GAPDH was used as markers for the cytoplasm, and the U6 were used as markers for the nucleus. B, Silver staining was performed using proteins pulled down by AFDN‐DT. The antisense sequence of AFDN‐DT was used as the negative primer. C, Illustration of AFDN‐DT directly interacted proteins

Next, to determine the mechanism underlying AFDN‐DT‐mediated growth and metastasis inhibition, we first determined the distribution of AFDN‐DT lncRNA in the cytoplasm and nucleus. We found that AFDN‐DT was mainly expressed in the nucleus, suggesting its potential function in transcriptional regulation (Figure 3A). Next, we performed an RNA pull‐down assay coupled with LC‐MS/MS to determine the interactome of AFDN‐DT in the HGC27 cells. As shown in Figure 3B, silver‐staining results of the proteins pulled down by AFDN‐DT suggested that AFDN‐DT can specifically bind to certain proteins. Moreover, the LC‐MS/MS results revealed 14 proteins that could directly interact with AFDN‐DT with high confidence (Figure 3C). We found that these target proteins were mainly involved in nucleosome organizations, such as HIST1H4A, HIST1H1C, HIST1H2BK and H3F3C. Notably, the identified targets were core histone proteins, suggesting the possible transcriptional regulatory function of AFDN‐DT in GC, and this needs to be explored in future studies.

### 3.4 Involvement of AFDN‐DT in transcriptional regulation through directly binding to the regulatory regions

**Figure 4:**
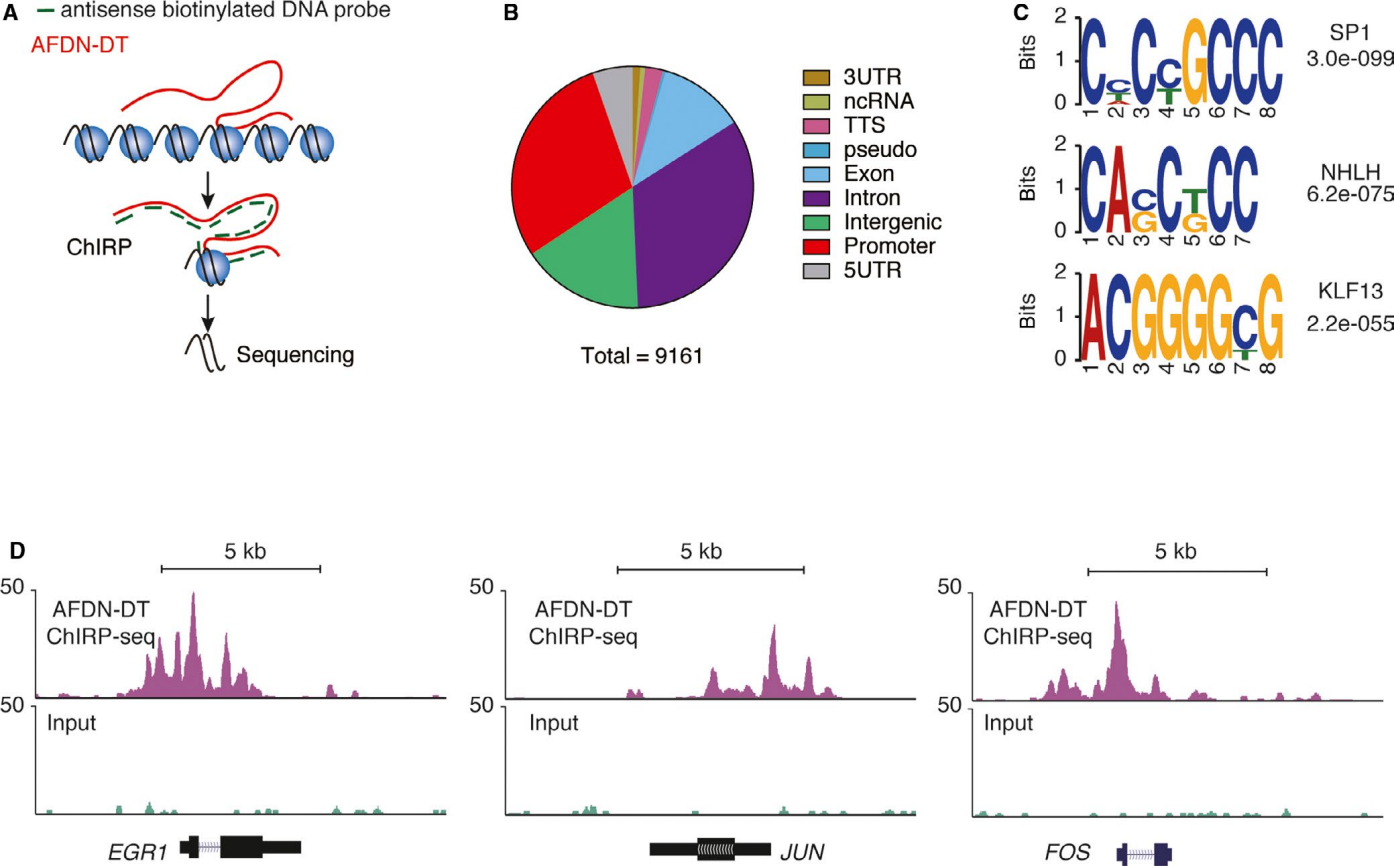
AFDN‐DT directly bound at the transcriptional regulatory regions. A, Schematic illustration of the ChIRP‐seq analysis. The AFDN‐DT was captured by antisense biotinylated DNA probes, and the AFDN‐DT interacted DNA was enriched and sequenced. B, Distribution of AFDN‐DT directs bound regions. A total of 9161 AFDN‐DT bound regions were annotated according to the genomic distribution. C, Motif enrichment of AFDN‐DT bound regions. D, Representative illustration of AFDN‐DT bound regions. The AFDN‐DT directly bounds at the promoter and gene body regions of EGR1, JUN and FOS

To investigate the direct targets of transcription regulation by AFDN‐DT, we performed the ChIRP using biotinylated antisense DNA probe sets (Figure 4A). We identified 9162 AFDN‐DT targets that directly bind to regions on the chromatin (Table S1). Of these regions, 29% were promoters, whereas 44.8% were regions on the gene (including exons and introns) (Figure 4B). Next, we performed motif enrichment analysis of these regions and found that the motifs, SP1, NHLH and KLF13, were significantly enriched (Figure 4C), suggesting that AFDN‐DT might affect the transcriptional function of these factors by regulating gene expression. As shown in Figure 4D, AFDN‐DT directly binds to the promoter of essential transcription factors, such as JUN, FOS and ERG1, indicating an important function of AFDN‐DT in gene regulation.

### 3.5 Involvement of AFDN‐DT regulated genes in transcriptional regulation

**Figure 5:**
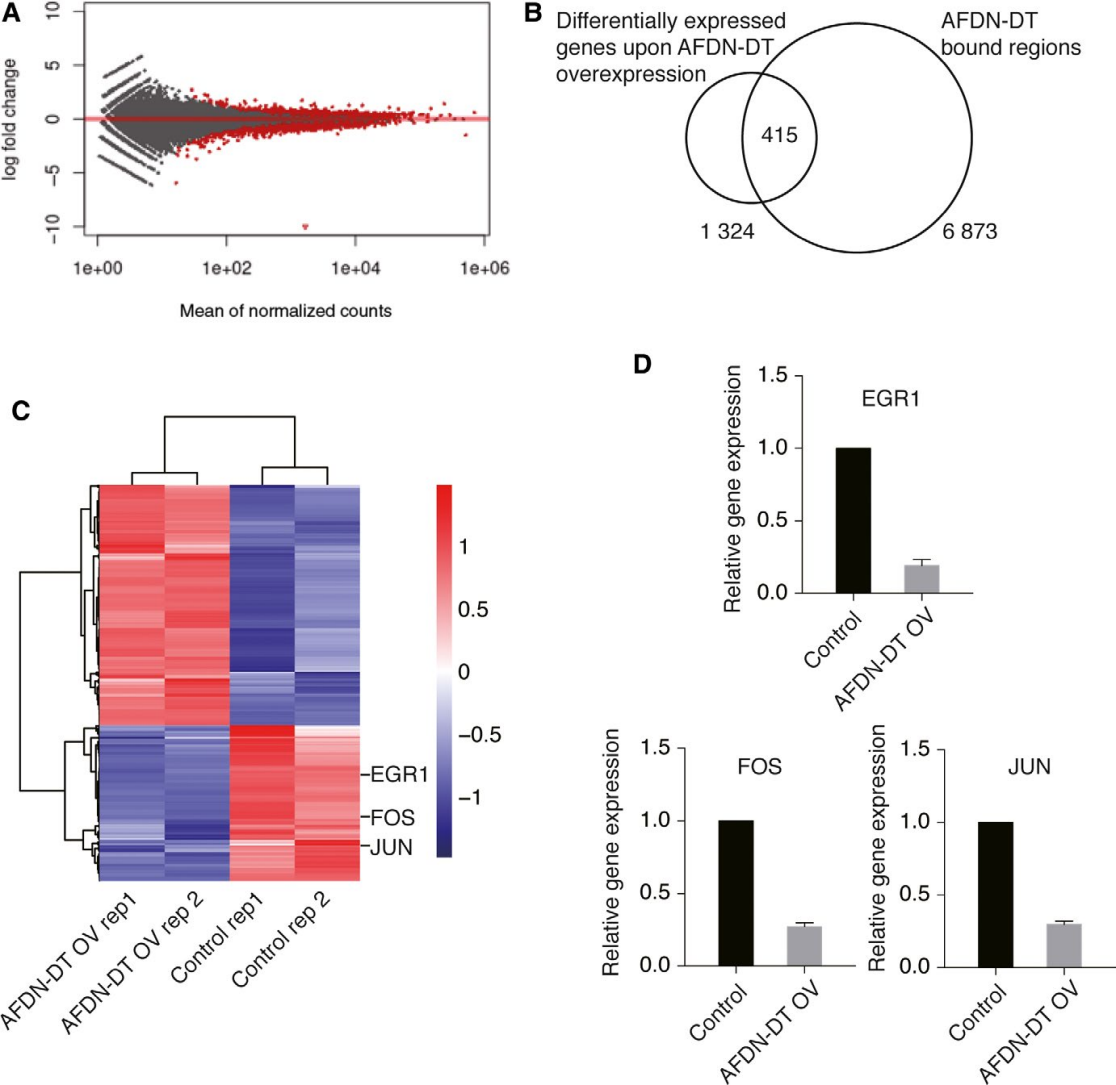
AFDN‐DT involves in the regulation of multiple pathways. A, RNA‐seq were performed to identify genes regulated by DN‐DT. Fold change and statistic p values in cells with or without AFDN‐DT overexpression were plotted. B, Identification of AFDN‐DT directly regulated target genes. The AFDN‐DT directly regulated target genes were defined by overlapping genes differentially expressed upon AFDN‐DT overexpression and genes directly bound by AFDN‐DT on chromatin. C, Heatmap showing genes directly regulated by AFDN‐DT. The EGR1, FOS and JUN were directly repressed by AFDN‐DT. D, RT‐qPCR validation of EGR1, FOS and JUN expression upon AFDN‐DT overexpression

To further understand the effects of AFDN‐DT on transcriptional regulation, we performed RNA‐seq experiments in HGC27 cells with or without AFDN‐DT overexpression (Figure 5A and Table S2). Following this experiment, we overlapped the genes that were differentially expressed in cells with AFDN‐DT overexpression, and the directly bound genes were identified using ChIRP‐seq. We identified 415 genes that were directly regulated by AFDN‐DT (Figure 5B‐C and Table S3). EGR1, FOS and JUN and their corresponding proteins were all down‐regulated by overexpression of AFDN‐DT (Figure 5C). This observation was further validated by RT‐qPCR using HGC27 cells with or without AFDN‐DT overexpression (Figure 5D). Furthermore, the gene ontology analysis revealed that the genes directly regulated by AFDN‐DT were mainly involved in transcriptional regulation (Table S4).

### 3.6 Repression of AFDN‐DT by DNA hypermethylation in GC

**Figure 6:**
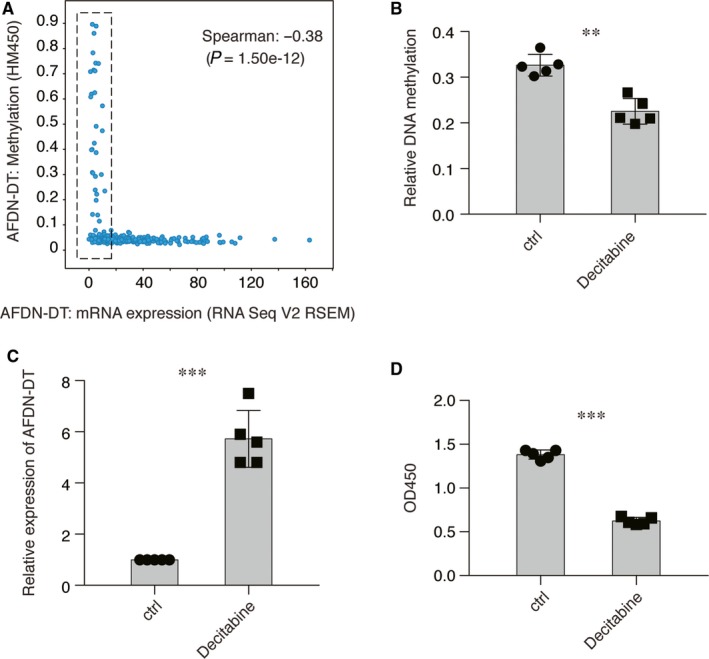
Down‐regulation of AFDN‐DT is contributed by DNA hypermethylation in gastric cancer. A, The expression of AFDN‐DT was negatively correlated with the DNA methylation of AFDN‐DT. The expression of AFDN‐DT and the normalized beta value in gastric cancer patient in TCGA cohorts were plotted. B, Decitabine decreased the DNA methylation on the promoter region of AFDN‐DT. The DNA methylation was determined by meDIP‐qPCR using antibodies against 5‐mC. Primers against the CpG island located at the promoter region of AFDN‐DT were used. C, DNA demethylation restored the expression of AFDN‐DT in HGC27 cells. Relative expression of AFDN‐DT was determined with or without Decitabine treatment. D, DNA demethylation induces HGC27 cell growth inhibition. Decitabine was used to inhibit the DNA methylation. Cell growth after 24 h treatment of Decitabine was determined by CCK8 assay. Data represent the mean of five replicates ± SD, **P < .001, ***P < .0001

To determine the mechanism underlying the deregulation of AFDN‐DT in GC, We analysed DNA methylation, which is an inheritable epigenetic modification, of the AFDN‐DT gene in patients with GC from TCGA cohorts.^29^ We found that the DNA methylation of AFDN‐DT negatively correlated with AFDN‐DT expression in the patients (Figure 6A). To confirm the direct link between AFDN‐DT DNA methylation and AFDN‐DT expression, we treated HGC27 cell lines with decitabine, a DNA methylation inhibitor, for 24 hours. Subsequently, we found that its expression was significantly restored upon inhibition of DNA methylation and the growth of HGC27 cells was significantly repressed (Figure 6B‐D). Taken together, these observations showed the mechanism underlying the down‐regulation of AFDN‐DT in GC.

## 4 DISCUSSION

LncRNA belongs to the largest category of RNAs and has been found to be frequently dysregulated in malignant diseases. Current studies on lncRNA mainly focus on their oncogenic role during malignant transformation and cancer progression. In the present study, we systematically investigated the function of AFDN‐DT in GC and demonstrated its role as a tumour suppressor through transcriptional regulation of the genes essential for malignant transformation.

Our previous studies demonstrated that AFDN‐DT was down‐regulated in GC, and this was associated with poor prognosis in patients with GC.^20^ However, we were unable to elucidate the function and mechanisms of AFDN‐DT in GC. In addition, a study showed that the expression of AFDN‐DT decreases in oral squamous cell carcinoma samples compared to the healthy oral mucosal cells.^30^ Here, we demonstrated that AFDN‐DT exerts tumour suppressive function in GC using both in vitro and in vivo models.

LncRNAs are regulators involved in multiple biological processes,^6^, ^8^ including microRNA sponging,^9^ translational 10 and transcriptional regulation^11^ and protein‐protein interactions.^12^ Recent studies on the function of lncRNAs have revealed the direct histone binding and transcriptional regulation functions of lncRNAs. For instance, MALAT1, which is an lncRNA, directly interacts with DBC1 and P53 and regulates the acetylation of P53 protein.^31^ Here, we demonstrated that AFDN‐DT also interacts with core histone proteins and directly binds to the transcriptional regulatory regions of the genes essential for malignant transformation, such as FOS, JUN and EGR1, which encode three of the most widely known transcriptional factors in cancer. The FOS and JUN belong to the AP‐1 transcriptional complex and have been reported to regulate the malignant transformation in GC.^32^, ^33^ We found that the AFDN‐DT overexpression down‐regulated the expression of FOS and JUN, indicating that AFDN‐DT directly inhibits the oncogenes in GC, and this further validates its tumour suppressive function.

Dysregulation of DNA methylation considerably influences gene expression, often resulting in malignant transformation.^34^ Many studies on genes coding for tumour suppressive proteins as well as microRNAs have established that DNA hypermethylation contributes to tumour suppression, which eventually leads to carcinogenesis in gastric tissues.^35^, ^36^ Here, we reported that DNA hypermethylation of the AFDN‐DT promoter down‐regulated the expression of AFDN‐DT in the tumour samples. Furthermore, we found that demethylation by decitabine increased the expression of AFDN‐DT. However, it is also possible that decitabine‐induced inhibition of cell growth might be due to the restored expression of other tumour suppressor genes. Therefore, the role of the AFDN‐DT‐mediated regulation network and its association with other tumour suppressive factors should be explored in the future.

## CONFLICTS OF INTEREST

The authors declare no conflicts of interest.

## AUTHOR CONTRIBUTION

YXL designed the study, performed experiments and wrote the manuscripts; JW, LW and CYM performed experiments; KX performed the bioinformatic analysis; PX designed the study, interpreted the results and wrote the manuscript. All authors read and approved the final manuscript.

## ETHICS APPROVAL AND CONSENT TO PARTICIPATE

The study was approved by the institutional research ethics committee of Songjiang Hospital Affiliated to Shanghai Jiaotong University School of Medicine (Preparatory Stage).

## Data Availability

The data sets used and/or analysed during the current study are available from the corresponding author on reasonable request.
